# Electrochemical Determination of Epinephrine in Pharmaceutical Preparation Using Laponite Clay-Modified Graphene Inkjet-Printed Electrode

**DOI:** 10.3390/molecules28145487

**Published:** 2023-07-18

**Authors:** Chancellin Nkepdep Pecheu, Victor Kougoum Tchieda, Kevin Yemele Tajeu, Sherman Lesly Zambou Jiokeng, Andreas Lesch, Ignas Kenfack Tonle, Emmanuel Ngameni, Christoph Janiak

**Affiliations:** 1 Electrochemistry and Chemistry of Materials, Department of Chemistry, University of Dschang, Dschang P.O. Box 67, Cameroon; chancellinpecheu@yahoo.fr (C.N.P.); victor.tchieda@univ-dschang.org (V.K.T.); tasergekev@yahoo.fr (K.Y.T.); sherman.jiokeng@univ-dschang.org (S.L.Z.J.); ignas.tonle@univ-dschang.org (I.K.T.); 2 Institut für Anorganische Chemie und Strukturchemie, Heinrich-Heine-Universität Düsseldorf, 40204 Düsseldorf, Germany; 3 Department of Industrial Chemistry “Toso Montanari”, University of Bologna, Viale del Risorgimento 4, 40136 Bologna, Italy; andreas.lesch@unibo.it; 4 Laboratory of Analytical Chemistry, Faculty of Science, The University of Yaounde 1, Yaounde P.O. Box 812, Cameroon

**Keywords:** epinephrine, laponite, cyclic voltammetry, inkjet-printed graphene electrode

## Abstract

Epinephrine (EP, also called adrenaline) is a compound belonging to the catecholamine neurotransmitter family. It can cause neurodegenerative diseases, such as Alzheimer’s disease, Parkinson’s disease, Huntington’s disease and amyotrophic lateral sclerosis. This work describes an amperometric sensor for the electroanalytical detection of EP by using an inkjet-printed graphene electrode (IPGE) that has been chemically modified by a thin layer of a laponite (La) clay mineral. The ion exchange properties and permeability of the chemically modified electrode (denoted La/IPGE) were evaluated using multi-sweep cyclic voltammetry, while its charge transfer resistance was determined by electrochemical impedance spectroscopy. The results showed that La/IPGE exhibited higher sensitivity to EP compared to the bare IPGE. The developed sensor was directly applied for the determination of EP in aqueous solution using differential pulse voltammetry. Under optimized conditions, a linear calibration graph was obtained in the concentration range between 0.8 µM and 10 μM. The anodic peak current of EP was directly proportional to its concentration, leading to detection limits of 0.34 μM and 0.26 μM with bare IPGE and La/IPGE, respectively. The sensor was successfully applied for the determination of EP in pharmaceutical preparations. Recovery rates and the effects of interfering species on the detection of EP were evaluated to highlight the selectivity of the elaborated sensor.

## 1. Introduction

Epinephrine (EP, also called adrenaline) is a molecule belonging to the catecholamine neurotransmitters family and is naturally synthesized in the human body from L-phenylalanine and L-tyrosine. EP is, in particular, found in the central nervous system of mammals, where it plays an important physiological role in muscle and tissue control but also as a chemical mediator for transmitting nerve impulses to efferent organs [1,2]. Its clinical applications include the treatment of hypertension, the treatment of serious allergic reactions, bronchial asthma, heartbeat stimulation and emergency medical situations [3]. EP is considered among the molecules that generally induce neurodegenerative diseases, such as Alzheimer’s, Parkinson’s and Huntington’s diseases, amyotrophic lateral sclerosis and frontotemporal dementia [4]. These neurodegenerative diseases are caused by an imbalance of neurotransmitters in the brain and body due to the damage or destruction of the nerve cells that control cognitive functions [5].

For the quantification of EP, several methods have been exploited that include chemiluminescence [6], capillary electrophoresis [7], liquid chromatography [8] and fluorimetry [9]. Although these methods are reliable and operate quite well, they meet, however, several drawbacks, which mainly include long analysis times, the complexity of sample pretreatment or preparation, high costs of equipment and the need for skilled operators. Electroanalytical methods, particularly using disposable printed electrodes and sensors, have shown their abilities to overcome many of these limitations for a wide range of analytes as they are rapid, simple and easy to handle at relatively low instrument and maintenance costs [10,11]. Electrochemical sensors are an integral part of modern analytical chemistry, converting the information associated with electrochemical reactions into an applicable qualitative or quantitative signal [12]. To date, several sensors have been proposed for the electrochemical determination of EP, as recently reviewed by Mattos et al. [13]. The quantification of EP in physiological media, as well as in pharmaceutical preparations, is attractive and of key importance in both analytical and biomedical sciences. Thus, there is a growing interest in electroanalytical chemistry for providing low-cost, sensitive and selective sensors for the detection of EP. In order to reduce costs and to develop disposable portable sensors for in-field and at-patient measurements, a growing interest in electroanalytical sensors for the low-cost, sensitive and selective detection of EP is noted in the literature. Some recent approaches to electrochemical EP sensors include a glassy carbon electrode modified either with gold nanoparticles and a cysteic acid film [14] or with an ordered mesoporous carbon/nickel oxide composite [15]. A screen-printed electrode was modified with a nanocomposite film consisting of electrochemically reduced graphene oxide and NiO nanoparticles to detect EP [16]. Graphene is a two-dimensional honeycomb-like carbonaceous material with a unique basal structure, charge transporter mobility, high thermal conductivity, broad electrochemical spectrum and excellent physicochemical properties. Its high specific surface area enables it to support a high load of biomolecules for good detection sensitivity [17].

Several previous studies on selective and sensitive electrochemical sensing have demonstrated the usefulness of clay minerals as electrode surface modifiers. Clay minerals can be used to increase the local analyte concentration on various solid electrodes due to their ordered structure formed mainly by aluminum phyllosilicate polyhedra organized in a two-dimensional stable network with intercalation ability to entrap certain guest molecules [18]. Laponite (La), for instance, is a synthetic clay mineral with a structure and composition close to that of the natural clay mineral hectorite [19]. Its disk-like shape has a thickness of 0.92 nm, an exchange capacity of 50–55 mmol/100 g and an average diameter of 25 nm, with a surface area of up to 370 m^2^·g^−1^. It bears a permanent negative surface charge on every single face and a positive charge distribution along the surface edges [20,21]. These properties make it an interesting electrode material, notably when the envisaged clay-modified electrode is designed for the electroanalysis of cationic species.

Taking into consideration the above-mentioned La features, we present here a simple and selective amperometric sensor for the electroanalysis and detection of EP in aqueous solutions and in pharmaceutical formulations. This was achieved by depositing a thin film of La on an inkjet-printed graphene electrode (IPGE). The charge properties and the conductivity of the obtained La/IPGE were characterized by using multi-sweep cyclic voltammetry (MCV) and electrochemical impedance spectroscopy (EIS). Important parameters likely to influence the voltammetric response of EP were optimized using differential pulse voltammetry (DPV). Finally, the selectivity of the proposed sensor was investigated, followed by its exploitation for the detection of EP in aqueous measurement solutions and in a real sample, i.e., a pharmaceutical formulation.

## 2. Results and Discussion

### 2.1. Electrochemical Characterizations of Non-Modified and La-Modified Inkjet-Printed Graphene Electrodes

**Figure 1:**
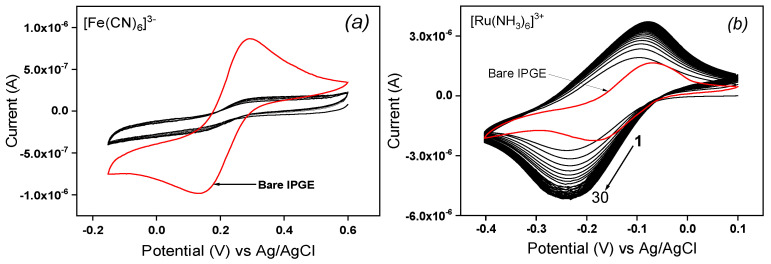
Multi-sweep cyclic voltammograms (30 scans) recorded on La/IPGE (black curves) in 0.1 M KCl containing (**a**) 1 mM [Fe(CN)_6_]^3−^ and (**b**) 1 mM [Ru(NH_3_)_6_]^3+^. The red curve represents the signal of the same redox probes on the bare IPGE. Potential scan rate: 30 mV/s.

The electrochemical characterization of the La clay mineral-modified inkjet-printed graphene electrode (La/IPGE) was first performed by MCV in a neutral pH solution containing respectively two redox probes ([Fe(CN)_6_]^3−^ and [Ru(NH_3_)_6_]^3+^). These redox species are generally considered for their known electrochemical behavior, and the changes in electrochemical responses correlated to the modification of electrode surfaces enable the development of an understanding of the observed processes. The studied potential ranges during MCV were from −0.2 V to 0.6 V and from −0.4 V to 0.2 V for [Fe(CN)_6_]^3−^ and [Ru(NH_3_)_6_]^3+^, respectively. For the 30 cyclic scans realized with La/IPGE in 0.1 M KCl containing ([Fe(CN)_6_]^3−^ (black curve in Figure 1a), a poor accumulation of the [Fe(CN)_6_]^3−^ redox probe was observed by the flat voltammograms. This was attributed to unfavorable electrostatic repulsion between the redox probe and the surface of the La sheets, both negatively charged under the applied experimental conditions. By replacing the negatively charged redox probe with the cationic and thus positively charged one (i.e., [Ru(NH_3_)_6_]^3+^), a progressive accumulation of the analyte was noticed (black curve in Figure 1b), which led to higher peak currents with the saturation of the electrode response from the 10th cyclic scan. This result is most likely due to the electrostatic attraction between the negatively charged La clay sheets and [Ru(NH_3_)_6_]^3+^. The electrochemical response of the same cationic probe was also investigated on the bare, i.e., unmodified, IPGE (red curve in Figure 1b). It can be observed that the presence of the La film on the IPGE increased the analyte signal and thus the sensitivity, as the current obtained upon signal saturation is more than three-fold higher on La/IPGE compared to the response on the unmodified electrode (i.e., IPGE). One can partially conclude that La/IPGE could be favorably applied for the voltammetric analysis of cationic species in 0.1 M KCl.

### 2.2. EIS Analysis of Bare IPGE and La/IPGE

**Figure 2:**
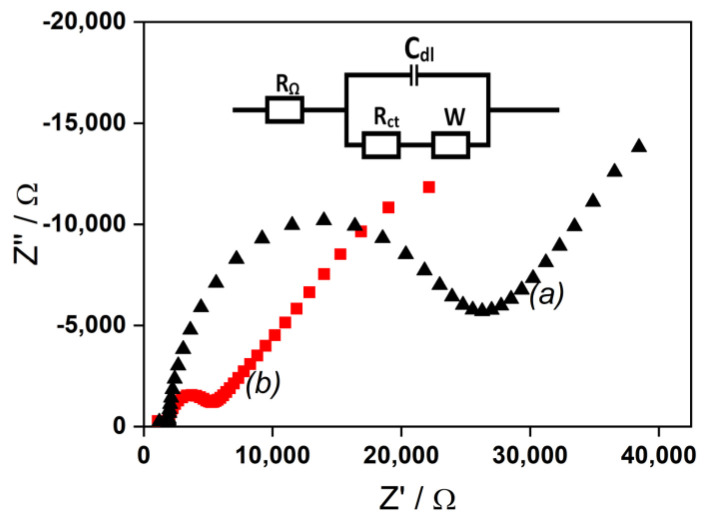
EIS recorded in 0.1 M KCl containing 1 mM [Fe(CN)_6_]^3−/4−^ (a) on La/IPGE (black data points) and (b) on bare IPGE (red data points).

The charge transfer resistance (R_ct_) of IPGE before and after its modification using the clay film was evaluated by EIS in the frequency range of 0.01 Hz to 10,000 Hz using [Fe(CN)_6_]^3−/4−^ in 0.1 M KCl. The corresponding Nyquist plots are shown in Figure 2.

The modified La/IPGE presented the largest semicircle with a charge transfer resistance of 2299.6 Ω. Oppositely, the unmodified IPGE gave the lowest semicircle in the region of low frequency with a charge transfer resistance of 1510.9 Ω. These observations are typical of electrochemical processes characterized by electron transfer. As obtained in the previous section, the electron transfer is limited with La/IPGE while favored on bare IPGE. Again, the behavior observed with La/IPGE can be explained by the best conductivity of the clay-modified electrode due to the electrostatic repulsion between complex ions [Fe(CN)_6_]^3−/4−^ and the negative sheets on laponite clay in accordance with the conclusion obtained in cyclic voltammetry.

### 2.3. Electrochemical Detection of EP on the IPGE Modified Electrode

#### 2.3.1. Electrochemical Behavior of EP by Cyclic Voltammetry

**Figure 3:**
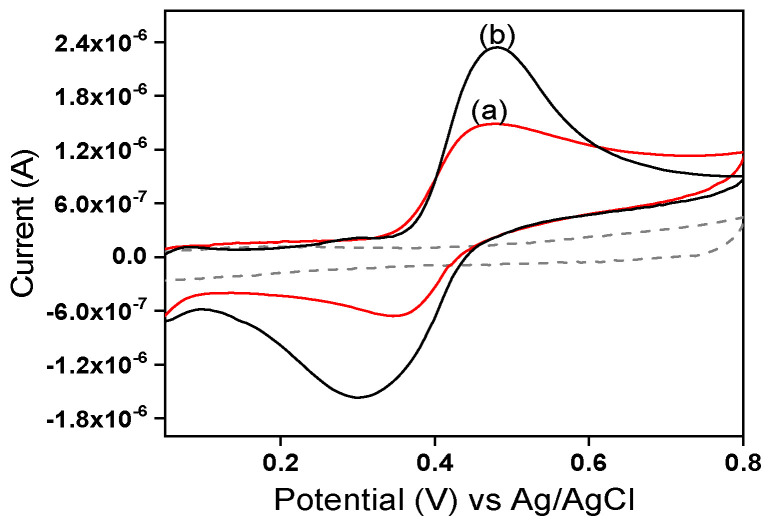
Cyclic voltammograms recorded in 0.1 M acetate buffer (AB) solution (pH 3.75) containing 0.4 mM EP, (a) on bare IPGE (red curve) and (b) on La/IPGE (black curve). The dotted line corresponds to the blank electrolyte. Potential scan rate: 50 mV/s.

Preliminary investigations were undertaken by using cyclic voltammetry in 0.1 M of acetate buffer solution (pH 3.75) to both study the electrochemical behavior of EP and evaluate the contribution of the laponite clay used to modify IPGE. The electrochemical response of EP was shown to be a quasi-reversible system on both simple IPGE and laponite clay-modified La/IPGE. Peaks were obtained at +0.47 V with a current of 1.27 µA in the anodic direction and at +0.35 V with an intensity of −0.71 µA in the cathodic direction for unmodified IPGE (Figure 3, curve (a)). Additionally, with laponite clay-modified IPGE, the signal occurs at +0.48 V with an anodic peak current of 2.02 µA and at +0.30 V with a reduction current peak of −1.65 µA (Figure 3, curve (b)).

Overall, it was noticed that the La/IPGE is more sensitive for EP compared to the bare IPGE. The presence of the clay mineral on IPGE has favored the retention of EP due to favorable interaction between the negatively charged clay platelets and the cationic form of EP.

#### 2.3.2. Influence of the Detection Medium

**Figure 4:**
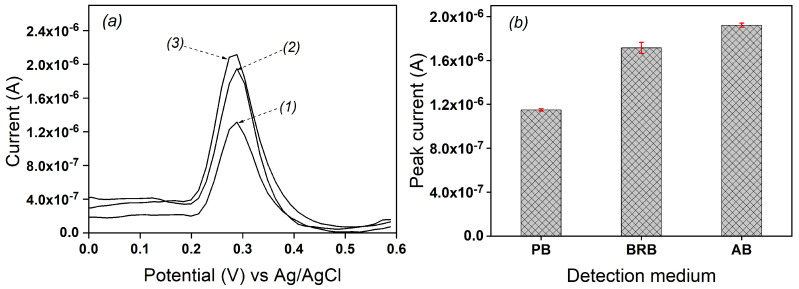
(**a**) DPV curves recorded on La/IPGE in (1) 0.1 M PB solution, (2) 0.1 M BRB solution and (3) 0.1 M AB solution (all at pH 5) containing 10^−4^ M EP, (**b**) corresponding peak currents.

Three media were tested to evaluate their individual influence on the electrochemical response of EP. Differential pulse voltammetry of 0.1 mM EP was recorded in 0.1 M acetate buffer (AB) solution, Britton Robinson buffer (BRB) solution and phosphate buffer (PB) solution, respectively, at pH 5 on the laponite clay-modified IPGE and the results are shown in Figure 4a. The peak current values of 1.23 µA, 1.721 µA and 1.949 µA were respectively obtained in PB, BRB and AB solution, as seen in Figure 4b. The highest and thus most favorable current value was obtained in AB solution. Consequently, the AB solution was retained for the further experiments.

#### 2.3.3. Influence of Scan Rate on the Electrochemical Response of EP on La/IPGE

**Figure 5:**
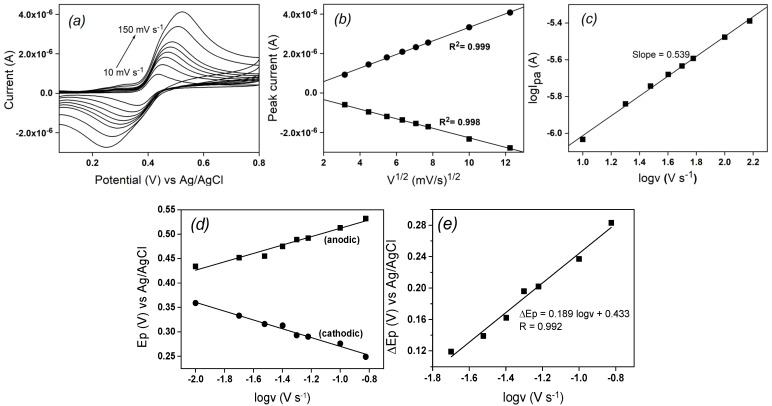
(**a**) CV curves recorded in 0.1 M AB solution (pH 3.75) containing 0.1 mM EP on La/IPGE at different scan rates: 10, 20, 30, 40, 50, 60, 100 and 150 mV s^−1^, (**b**) peak current of both anodic and cathodic vs. square root of scan rate, (**c**) logI_pa_ vs. logν, (**d**) E_p_ vs. logν and (**e**) ΔE_p_ vs. logν.

The influence of the scan rate on the oxidation and reduction peaks of EP was studied using cyclic voltammetry in AB solution (pH 3.75) on La/IPGE, and the obtained results are shown in Figure 5. It was observed that increasing the scan rate ν from 10 to 150 mV s^−1^ induced an increase in both anodic and cathodic peak currents of EP.

Meanwhile, the anodic signal shifted towards more positive potential values and cathodic peaks to more negative potential values (Figure 5a), in tandem with an increase of the ΔE_p_ value, indicating a quasi-reversible process. By plotting both anodic and cathodic peak currents as a function of the square root of the scan rate in the studied range, a linear dependence was observed (Figure 5b), indicating a diffusion-controlled process.

This fact was confirmed by also plotting the logarithmic of anodic peak current versus the logarithmic of the scan rate (Figure 5c): a linear dependence was again obtained between both parameters, according to the following equation logI_pa_ (µA) = −6.552 + 0.539 logν (V s^−1^) (R^2^ = 0.997). The slope value of 0.54, close to the standard theoretical value of 0.5, confirmed the pure diffusion-controlled process [22]. The peak potential (E_p_) and the logarithm of the scan rate showed a linear relationship (Figure 5d), also indicating that the electrocatalytic oxidation of EP on the modified electrode surface is a typical quasi-reversible process. Two linear regression equations were derived from the plots: E_pa_ (V) = 0.086 logν (V s^−1^) + 0.598 (R = 0.983), and E_pc_ (V) = − 0.090 logν (V s^−1^) + 0.180 (R = − 0.992). According to Laviron’s equation [23], plotting the peak potential versus the logarithm of the scan rate yields two straight lines with slopes of 2.3RT/(1- α)nF and −2.3RT/αnF for the anodic and cathodic peaks, respectively. The charge transfer coefficient (α = 0.5) was calculated from the slopes. The apparent heterogeneous electron transfer rate constant (k_s_) can be obtained based on Equation (1):

(1) $${\mathrm{logk}}_{s}=\alpha \log(1-\alpha )+(1-\alpha )\log\alpha -\log(\mathrm{RT}/\mathrm{nFv})-\left[(1-\alpha )\alpha \mathrm{nF}/2.3\mathrm{RT}\right]∆E_{p}$$

where n is the number of electrons involved in the reaction, ΔE_p_ is the peak potential separation (E_pa_ − E_pc_ = ΔE_p_ ˃ 200/n mV), ν is the scan rate, and all other symbols have their conventional meanings. From the values of ΔE_p_ corresponding to different sweep rates (Figure 5e), an average value of k_s_ of 4.85 s^−1^ was found.

#### 2.3.4. Influence of pH on the Electrochemical Response of EP

**Figure 6:**
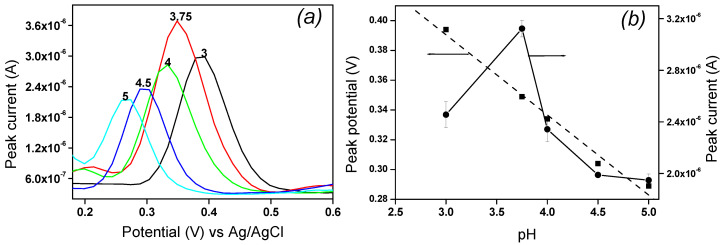
(**a**) DPV curves recorded on La/IPGE for 10^−4^ M EP in 0.1 M AB solution at different pH values (3, 3.75, 4, 4.5 and 5); (**b**) EP anodic peak current and peak potential vs. pH.

The redox behavior of EP implies protons, and the influence of pH on its electrochemical response was studied from 3 to 5 in 0.1 M AB solution containing 10^−4^ M EP by DPV. The results are shown in Figure 6. In the study range (pH 3 to 5), high EP oxidation peak currents are observed, with a maximum reached at pH 3.75, followed by a slight decrease for pH values between 3.75 and 5 (Figure 6a). The slight increase in the peak current between pH 3 and 3.75 is due to the fact that in this range, EP exists mainly in its cationic form (pKa = 8), as shown in the distribution diagram drawn up by Corona et al. [24]. Thus, the main affinity between the cationic form of EP and the negative charge of laponite is electrostatic attraction.

**Scheme 1:**
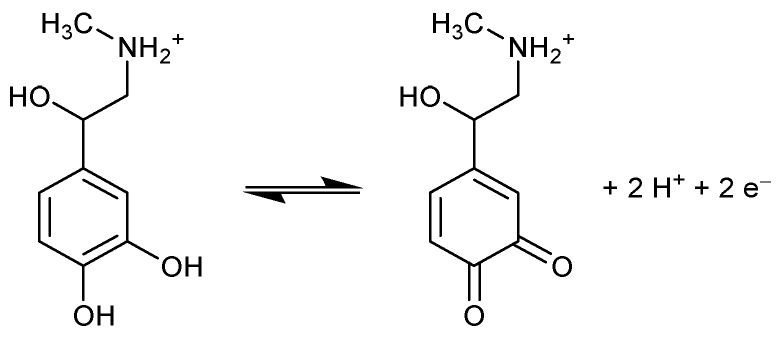
The proposed electrochemical redox reaction of EP [25].

From pH 4 to 5, peak currents decrease progressively. Although the laponite surfaces remain negatively charged and the EP positively charged, this decrease in peak current can be attributed to the decreasing acidity of the medium, thereby weakening the interaction between EP and laponite. In addition, the peak potential of EP shifted to more negative values and remained linearly on the pH values range studied, as shown in Figure 6b. The obtained anodic linear regression equation was E_pa_ (V) = −0.054 pH + 0.574, and the correlation coefficient was R^2^ = 0.986. The slope value of −0.054 V/pH near the Nernstian value of 0.059 suggested that an equal number of protons and electrons is exchanged during electrochemical oxidation/oxidation of EP to epinephrinequinone on the La/IPGE sensor surface according to Scheme 1, as previously observed by Hu et al. [25].

#### 2.3.5. Influence of EP Concentration and Calibration Curve

**Figure 7:**
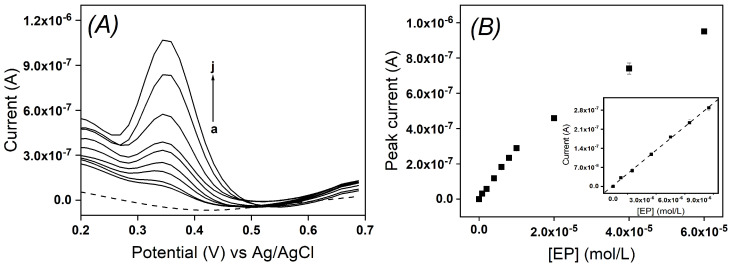
(**A**) DPV recorded at La/IPGE in 0.1 M AB solution (pH 3.75) containing EP at varied concentrations: (a) 0 µM, (b) 0.8 µM, (c) 2 µM, (d) 4 µM, (e) 6 µM, (f) 8 µM, (g) 10 µM, (h) 20 µM, (i) 40 µM and (j) 60 µM. (**B**) Plot of I**_p_** vs. [EP] (inset: calibration curve of EP in selected linear range).

The influence of the concentration of EP at La/IPGE in 0.1 M of AB solution studied by DPV is shown in Figure 7A. The peak current (I_p_) increased with the increase of EP concentration in the range of 0.8 µM to 60 μM (Figure 7B). The linearity was obtained between 0.8 and 10 μM, the corresponding calibration curve for which is shown in Figure 7B, which is described by the following Equation (2):

I_p_ = 0.02871 [EP] + 3.80835 × 10^−9^ (R^2^ = 0.9987) (2)

The detection limit, which is given by the relation 3S_b_/m where S_b_ represents the standard deviation obtained from the blank and m the slope on the calibration plot [26], was 0.26 µM. Under the same conditions, the detection limit of bare IPGE was estimated as 0.34 µM.

This result showed that La/IPGE could allow the detection of EP in the micromolar range in an aqueous solution. The detection limits obtained in this work were compared with other data based on modified electrodes, as gathered in Table 1. For the same (electrochemical) methods, one can notice that the detection limit obtained on La/IPGE (0.26 µM) is on the same order of magnitude or even better than those obtained in some previously reported works [27,28].

Globally, results obtained in this section indicated a good sensitivity of the La/IPGE sensor toward the detection of EP and that this sensor could serve as a low-cost analytical device. However, before applying it for the quantification of EP in a real sample, the effect of potential compounds expected to interfere with the signal of EP was evaluated.

### 2.4. Interference Study

**Figure 8:**
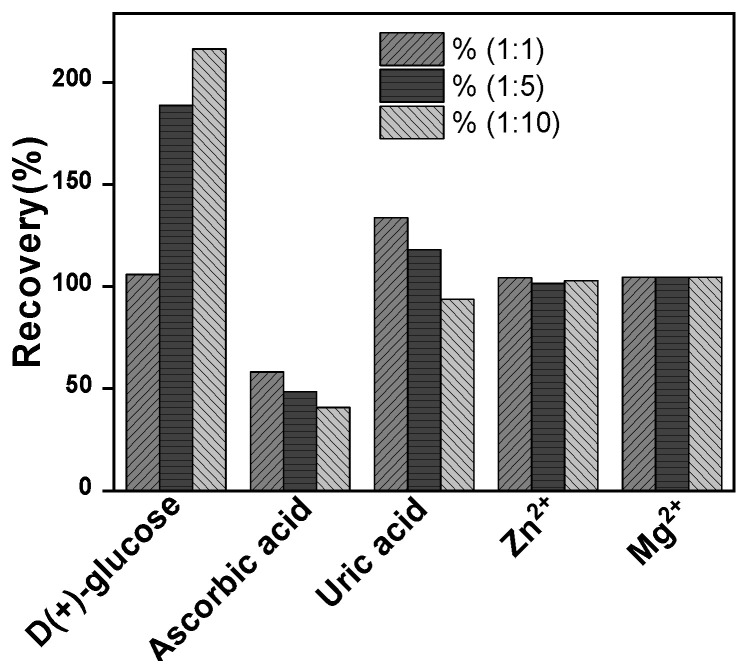
Influence of some potential interfering compounds on the DPV response of 0.1 mM EP with La/IPGE.

The selectivity of the La/IPGE sensor on the oxidation peak current of EP was evaluated by analyzing the effect of the presence in the same medium of EP, along with these compounds. They included the organic compounds ascorbic acid, uric acid, D-(+)-glucose, and two metal ions (Zn^2+^ and Mg^2+^) introduced in the supporting electrolyte at three different concentration ratios, as presented in Figure 8. It was noticed that neither Zn^2+^ nor Mg^2+^ ions influence the peak current of EP for the three investigated concentrations. However, ascorbic acid, uric acid and D-(+)-glucose significantly interfere with the EP signal.

This observation is due to the preferential occupation of both ascorbic and uric acid on the clay film on the IPGE surface, notably when the concentration of interfering species was increased. These observations somewhat limit the selectivity of the La/IPGE sensor and indicate that it should be used in media not containing the studied organic compounds.

### 2.5. Analytical Application

The analytical application of the La/IPGE-based EP sensor was demonstrated by using the sensor for the analysis of adrenaline in a commercial dose manufactured by Zhenjiang Tianfeng Pharmaceutical (Zhenjiang, China).

The sample was firstly diluted 100 and 400 times using the 0.1 M acetate buffer (AB) solution supporting electrolyte, with the final pH adjusted at 3.75. An appropriate volume of the solution to be tested (250 µL) was added to the electrochemical cell containing the electrolyte solution (25 mL, acetate buffer solution pH 3.75), and the peak current was measured. Using the calibration graph, the concentration in the solution was calculated. The data collected are shown in Table 2.

Good recovery rates were obtained, revealing that the proposed sensor could serve for the electrochemical determination of EP in real samples.

The recovery rates obtained were within our set limits, revealing, therefore, that the proposed sensor could indeed serve for the electrochemical determination of EP in real pharmaceutical samples.

## 3. Conclusions

This work was devoted to the preparation of an electrochemical sensor based on an inkjet-printed graphene electrode (IPGE), modified by a thin film of laponite (La) clay mineral. It was characterized by multi-sweep cyclic voltammetry and electrochemical impedance spectroscopy and then applied for the sensitive analysis of EP in an aqueous solution. Under optimal analysis conditions, the sensor provided detection limits of 0.34 µM and 0.26 µM with bare IPGE and La/IPGE, respectively. The effect of some potential interfering species has shown that both zinc and magnesium ions influence the peak current of EP, but ascorbic acid, uric acid and D-(+)-glucose significantly interfere with the electrochemical response of EP. Best recoveries were also obtained with the sensor after applying it for the determination of EP in pharmaceutical preparation. The obtained results show that this sensor could serve as an alternative tool for the determination of EP not only in aqueous solution but also in a pharmaceutical preparation with good sensitivity.

## 4. Experimental Section

### 4.1. Chemicals, Reagents and Materials

All chemicals were used without further purification. Epinephrine hydrochloride used as the target analyte was purchased from Across Organics. The stock solution of EP was prepared at 10^−2^ M by dissolving 9 mg of epinephrine hydrochloride in 5 mL of PB solution (pH 7) unless otherwise described. Working solutions of EP were prepared by dilution. Various buffer solutions were prepared using the following chemicals: Boric acid (>99%, Sigma-Aldrich, Saint Louis, MO, USA), acetic acid (≥99.7%, Fluka, Jinshangsheng, Shanghai, China) and phosphoric acid (85%, Sigma-Aldrich) for Britton Robinson buffer solution; Na_2_HPO_4_.2H_2_O (98%, Fluka) and KH_2_PO_4_ (99%, Analar, Princeton, NJ, USA) for phosphate buffer solution and sodium acetate (≥99.0%, Sigma-Aldrich) and acetic acid (≥99.7%, Alfa, Binfiedl, UK) for acetate buffer solution. The pH of buffer solutions used was adjusted by using decimolar solutions of HCl and NaOH (Merck, Darmstadt, Germany). D-(+) glucose, ascorbic acid; uric acid was obtained from Sigma-Aldrich (Taufkirchen, Germany); potassium hexacyanoferrate (III and II) and KCl from Prolabo (99%, Bern, Switzerland), Zn(NO_3_)_3_·6H_2_O from BDH chemicals Ltd. (99%, Poole, UK) and Mg(NO_3_)_3_·6H_2_O from Kermel (99%, Tianjin, China).

Laponite clay with the chemical formula Na_0.7_Si_8_Mg_5.5_Li_0.3_O_20_(OH)_4_ [31] used in this work was purchased from the Source Clay Repository Clay Mineral Society, Purdue University, USA. It was purified by collecting the fraction with particle size < 2 µm based on the Stokes law [32].

### 4.2. Preparation of Inkjet-Printed Graphene Electrode (IPGE)

**Figure 9:**
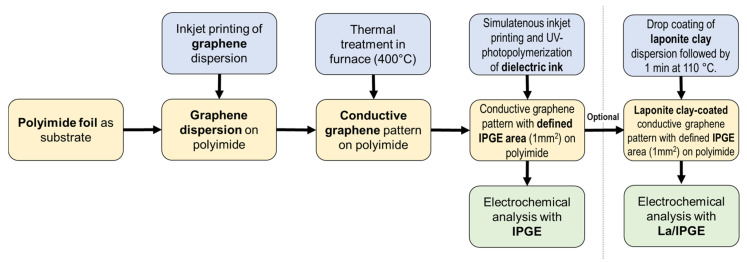
Workflow of production of the unmodified (IPGE) and modified (La/IPGE) inkjet-printed graphene electrode.

The inkjet-printed graphene electrode was made according to our previously published work (see the workflow in Figure 9) [33]. First, layers of the graphene dispersion were printed on polyimide (PI) foil (150 µm thickness, Dr. Dietrich Müller GmbH, Germany) using the DMP-2831 inkjet printer from Fujifilm Dimatix. This was followed by thermal post-processing in a furnace at 400 °C. Thereafter, the inkjet printer was combined with a Hg arc lamp (Omnicure S2000, Excelitas Technologies, Pleasanton, CA, USA) to simultaneously print and photo-polymerize the UV curable dielectric ink EMD 6200 (Sun Chemical, Parsippany-Troy Hills Township, NJ, USA) for defining the geometric surface area of the electrode to 1 mm^2^. These IPGEs were then used as fabricated without the need for further treatments. The optional chemical modification of the surface of the IPGE to fabricate the selective electrochemical sensor based on La/IPGE was then carried out by drop-coating using a laponite clay dispersion. For preparing the laponite clay dispersion, 5 mg of laponite (<2 µm particle size) clay was introduced in 1 mL of distilled water and dispersed by ultrasonication in an ultrasonic bath for 20 min. A volume of 0.5 µL of clay dispersion, a volume verified to match and fully coat the theoretical surface area (1 mm^2^) of the IPGE, was deposited on the active surface of IPGE and dried for 1 min at 110 °C on steam before its use for electrochemical investigations.

### 4.3. Apparatus

The electrochemical measurements were realized by using a µ-Autolab potentiostat (type III) running with GPES software Autolab 4.9 and with a standard three-electrode cell configuration. Voltammetric responses were recorded in supporting electrolyte with an Ag/AgCl/3M KCl electrode serving as reference electrode (RE), a steel rod serving as counter electrode (CE) and either a bare, i.e., unmodified, IPGE or chemically modified, i.e., La/IPGE, working electrode (WE). Cyclic voltammetry (CV) and differential pulse voltammetry (DPV) of known concentrations of adrenaline were recorded in 0.1 M of phosphate buffer (PB) solution, Britton Robinson buffer (BRB) solution and acetate buffer (AB) solution at known pH values in the potential range of 0 to 0.8 V. The pH values of solutions were measured and monitored with an Inolab pH meter at room temperature. Additionally, both electrochemical impedance spectroscopy (EIS) and cyclic voltammetric techniques were used for electrochemical characterization of unmodified and chemically modified IPGE. For EIS measurements, 0.1 M KCl containing 1 mM [Fe(CN)_6_]^3−/4−^ was used as the measurement solution, and the applied frequency range was 0.01 Hz to 10,000 Hz. For multi-sweep cyclic voltammetry (MCV), 0.1 M KCl containing either 1 mM [Fe(CN)_6_]^3−^ or 1 mM [Ru(NH_3_)_6_]^3+^ was used for the investigation of the effect of the surface change of La/IPGE on electrochemical responses.

## Figures and Tables

**Table 1:** Compared performance of selected sensors towards the detection of EP ^(a)^.

Modified Electrodes	Concentration Range (µM)	Detection Limit (µM)	Supporting Electrolyte (pH)	Methods	Ref.
GCE/Na[RuL_2_]	3–136	3.49	0.1 M PB (pH 7.5)	FIA	[27]
ECS/Na[RuL_2_]/cellulose acetate	3–273	0.289	0.1 M PB (pH 7.5)	FIA	[27]
Poly(EBT)-modified GCE	2.5–50	0.3	0.1 M PB (pH 3.5)	DPV	[28]
Iron(III) doped zeolite-modified CPE	0.9–216	0.44	0.1 M PB (pH 3.0)	DPV	[29]
Caffeic acid-modified GCE	2–300	0.60	0.15 M PB (pH 7.4)	CV	[30]
Bare IPGE	0.8–10	0.34	0.1 M AB (pH 3.75)	DPV	This work
Laponite clay-modified IPGE	0.8–10	0.26	0.1 M AB (pH 3.75)	DPV	This work

^(a)^ FIA: Flow Injection Amperometry, CPE: Carbon Paste Electrode, GCE: Glassy Carbon Electrode, Na[RuL_2_]: Ru(III) Schiff-base complex, IPGE: Inkjet-Printed Graphene Electrode, DPV: Differential Pulse Voltammetry, EBT: Eriochrome Black T, CV: Cyclic Voltammetry, AB: Acetate Buffer solution, PB: Phosphate Buffer solution.

**Table 2:** Electrochemical quantification of EP in a real sample.

Sample	Dilution Rate	[EP] Added (µM)	[EP] Founded (µM)	Recovery (%)
1	100	54.58	51.45	94.26
2	400	18.19	20.34	102.4

## Data Availability

Not applicable.

## Sample Availability

Sample of the modified laponite is available from the authors.
